# Long-term development of post-traumatic stress symptoms and associated risk factors in military service members deployed to Afghanistan: Results from the PRISMO 10-year follow-up

**DOI:** 10.1192/j.eurpsy.2020.113

**Published:** 2020-12-21

**Authors:** Sija J. van der Wal, Eric Vermetten, Geuze Elbert

**Affiliations:** 1 Brain Research and Innovation Centre, Ministry of Defence, Utrecht, The Netherlands; 2 Department of Psychiatry, Leiden University Medical Center, Leiden, The Netherlands; 3 UMC Utrecht Brain Center, Department of Psychiatry, University Medical Center Utrecht, Utrecht, The Netherlands; 4 Military Mental Healthcare, Ministry of Defence, Utrecht, The Netherlands

**Keywords:** Cohort study, deployment, latent trajectory analysis, longitudinal study, PTSD

## Abstract

**Background:**

Symptoms of post-traumatic stress disorder (PTSD) can manifest several years after trauma exposure, and may impact everyday life even longer. Military deployment can put soldiers at increased risk for developing PTSD symptoms. Longitudinal evaluations of PTSD symptoms in deployed military personnel are essential for mapping the long-term psychological burden of recent operations on our service members, and may improve current practice in veterans’ mental health care.

**Methods:**

The current study examined PTSD symptoms and associated risk factors in a cohort of Dutch Afghanistan veterans 10 years after homecoming. Participants (*N* = 963) were assessed seven times from predeployment up to 10 years after deployment. Growth mixture modeling was used to identify distinct trajectories of PTSD symptom development.

**Results:**

The probable PTSD prevalence at 10 years after deployment was 8%. Previously identified risk factors like younger age, lower rank, more deployment stressors, and less social support were still relevant 10 years after deployment. Four trajectories of PTSD symptom development were identified: resilient (85%), improved (6%), severely elevated-recovering (2%), and delayed onset (7%). Only the delayed onset group reported increasing symptom levels between 5 and 10 years postdeployment, even though 77% reported seeking help.

**Conclusions:**

This study provides insights into the long-term burden of deployment on the psychological health of military service members. It identifies a group of veterans with further increasing PTSD symptoms that does not seem to improve from currently available mental health support, and underlines the urgent need for developing and implementing alternative treatment opportunities for this group.

## Introduction

With over 25,000 troops deployed during 2005–2011, the Dutch participation in the International Security Assistance Force (ISAF) in Afghanistan was the first time the Dutch armed forces conducted a military mission of this size and complexity. In addition to the service members who lost their lives or suffered serious injuries during combat actions, the mission also left its psychological marks. As historical military conflicts teach us, signs of post-traumatic stress disorder (PTSD) can manifest several years or sometimes decades after the actual traumatic exposure, and may impact everyday life even longer [1]. Longitudinal, long-term evaluations of PTSD symptoms in this recently deployed group of military personnel are essential for mapping the psychological burden of recent operations on our service members, which may improve current practice in veterans’ mental healthcare and inform policymaking in future missions.

Different coalition partners have reported on the prevalence of PTSD in their deployed troops [2,3]. The pool of longitudinal studies that assessed military personnel on multiple time points is on the other hand less extensive, and available studies often ran for a limited period of time. Studies in U.S. National Guard soldiers [4] and in U.K. [5] and Dutch [6] armed forces deployed to Iraq or Afghanistan suggest a trend of stabilizing or aggravating PTSD prevalence rates in service members deployed in recent military missions, and underline the importance of long-term monitoring of the mental health of deployed personnel. Despite the importance of prevalence rates for expressing the impact of deployment on the psychological wellbeing of a whole military population and assessing treatment demands after homecoming, prevalence rates do not reflect the large heterogeneity in symptom development that exist between individuals. This heterogeneity can be addressed with the use of latent growth mixture modeling (LGMM) techniques. Recent longitudinal studies in military populations have utilized this approach, and identified distinct but overlapping trajectories of PTSD symptom development over time. Several studies report a three-class solution, but the shape of the trajectories vary and include resilient, improving, deteriorating, or chronic trajectories [4,6,7]. U.S. studies based on data of the Millennium Cohort, a large sample of U.S. active duty and reserve forces, are consistent in reporting a four-class solution involving a resilient, decreasing, increasing, and high symptom trajectory [8–10].

Beyond the traumatic experience itself, individual vulnerability factors can contribute to changes in PTSD symptom levels and developmental trajectories. Female gender, younger age, combat exposure, or previous trauma exposure are frequently identified as risk factors for combat-related PTSD [5,6,11]. Only a few studies aimed to identify vulnerability factors related to developmental trajectories of PTSD [4,7–10]. Factors related to increases in PTSD symptom levels after deployment can help to identify who is most at risk for developing PTSD symptoms, even after the acute phase of trauma, and target follow-up screening accordingly.

In the current study, we report on findings from the 10-year follow-up measurement in the PRISMO cohort, a large cohort of Dutch military personnel deployed to Afghanistan [12]. Previous trajectory studies did not include a predeployment measurement [10], had short follow-up times no longer than 3 years [4], or included only a few follow-up measurements [7–9]. We extended this research by studying the effects of deployment on PTSD symptoms on the long term, using a unique follow-up period of 10 years with seven consecutive measurement points. We aimed to identify trajectories of PTSD symptom development and assessed the role of different covariates on the development of PTSD symptoms. We hypothesized that the probable 10-year PTSD prevalence would significantly decline compared to 5-year after deployment. Based on the three trajectories identified in our 5-year follow-up report [6], we predicted a three-class solution with a resilient trajectory, a recovered trajectory, and a delayed onset trajectory that show symptom improvement between 5- and 10-years postdeployment.

## Methods

### Study design and participants

The present study is part of a large prospective cohort study on the development of stress-related mental health symptoms in deployed Dutch military personnel, the PRISMO study, which is described in detail elsewhere [12]. Recruitment resulted in the inclusion of 1,007 study participants, who were deployed for about 4 months in behalf of ISAF between March 2005 and September 2008. The baseline measurement was carried out approximately 1 month before deployment at the army base. The first two follow-up measurements were also completed at the army base, at approximately 1 and 6 months after the soldiers returned home. The 1-, 2-, and 5-year follow-up assessments were completed at home, and the 10-year follow-up was conducted at the research facility of the Military Mental Healthcare. All measurements consisted of paper-and-pencil questionnaires, except for the 5-year follow-up, which consisted of an online questionnaire. Written informed consent was obtained from all subjects. All procedures were approved by the Institutional Review Board of the University Medical Centre Utrecht (Utrecht, The Netherlands), approval number 01/333-0.

### Measures

#### PTSD symptoms

For all assessments symptoms of PTSD were measured with the Self-Rating Inventory for PTSD (SRIP) [13], a Dutch questionnaire to assess PTSD symptoms in the past 4 weeks based on the DSM IV criteria for PTSD. The SRIP contains 22 questions with responses measured on a Likert scale ranging from 1 (never) to 4 (very frequent). A higher sum score indicated more symptoms (range 22–88). The SRIP showed good internal consistency, discriminant validity, and concurrent validity with other commonly used PTSD measures [13,14]. As recommended in the literature, a cut-off score of 38 was used to indicate substantial PTSD symptoms [13,15].

#### Covariates

At baseline, participants provided information about their sex, age, educational level, rank, and previous deployments. More detailed information on the measurement scales of demographic information can be found in the Supplementary Materials. Potential traumatic experiences before the age of 18 were also assessed at baseline using the Early Trauma Inventory Self Report-Short Form (ETISR-SF), a questionnaire containing 27 items, of which the total sum represents the total number of different potential traumatic events experiences [16]. At the first measurement after deployment, information on the participant’s role during the mission was collected and divided in three categories: inside the base (function was exclusively carried out inside the military base; for example, logistics or medical work in the field hospital), outside the base (function was carried out outside the base; e.g., patrols), and both inside and outside the base (function included activities inside the base as well as outside the base). Their exposure to traumatic stress during deployment was assessed using the Deployment Experience Scale (DES), a 19-item deployment stressors checklist [17]. At all follow-up measurements, potential new deployments after the initial deployment at study inclusion were assessed. At the 1-year follow-up, social support during and after deployment were measured with the Deployment Risk and Resilience Inventory 1 (DRRI-1), a collection of measures for studying deployment-related experiences of military veterans [18]. Part F (support from other military personnel during deployment) and part L (support from family and friends after deployment) consists respectively of 12 and 15 items with responses on a Likert scale ranging from 1 (strongly disagree) to 5 (strongly agree), where higher scores indicated more received support.

#### Mental health support

The receipt of psychological care was assessed by the item “Have you ever received any care for psychological health complaints after your deployment?” at the 10-year follow-up measurement (see Supplementary Material).

### Statistical analysis

We assessed the change in PTSD symptom level at 10 years after deployment relative to the predeployment level in a mixed model analysis. The time variable was recoded into six dummy variables, one dummy variable for each measurement after deployment, whereby predeployment served as the reference. Continuous, longitudinal PTSD symptom scores at all seven measurements were used as the outcome variable. Covariates were included separately in the mixed models. Participants were included in the analyses if they had a PTSD assessment at one or more time points. A two-tailed *p* value of less than 0.05 was considered statistically significant.

LGMM analyses were conducted in Mplus version 8.4 to identify trajectories of PTSD symptom development. Latent class growth analysis (LCGA) as well as growth mixture modeling (GMM) were performed to identify the best performing model [19]. The models were re-fitted with a quadratic term for time to assess whether nonlinear growth curves provided better fit to the data. The models reflected the number of months between the different assessments. Missing data over time in the outcome variable was handled by full information maximum likelihood estimation. Missing values in the covariates were handled by multiple imputation. All models were compared on fit indices, entropy, class size, and interpretability. The percentage of participants that received psychological treatment was calculated for each trajectory. The effect of covariates on the trajectory assignment was investigated in adjusted multinomial regression models, in which the class assignment output from the LGMM analysis was the outcome variable. A three-step approach was used to account for the classification error of belonging to trajectory classes [20]. Details about the trajectory analysis are described in the Supplementary Materials.

## Results

Between 2005 and 2008, a total of 1,032 participants signed up for participation to the PRISMO study prior to deployment. Twenty-five participants were eventually not deployed, leaving a total of 1,007 study participants. Of those participants, 44 had no PTSD measurement at any of the time points and were excluded from the analyses. The baseline characteristics are shown in Table 1. Compared to participants without a PTSD measurement, participants with a PTSD measurement were more frequently deployed in 2007/2008 compared to 2005/2006 (*p* < 0.0001) and had a lower early trauma score (*p* = 0.001) (see Table 1).

**Table 1.** Demographics and other characteristics of participants in the cohort who were deployed, separated for participants included in the mixed model and latent trajectory analyses and participants with missing outcome values.

	Participants with outcome values at one or more time points (*n* = 963)^a^	Participants without any outcome values (*n* = 44)^a^	*p*-value
Sex			
Male	878 (91%)	43 (98%)	0.128
Female	85 (9%)	1 (2%)	
Age (years)^b^			
<21	130 (14%)	9 (23%)	0.091
≥21	831 (87%)	30 (77%)	..
Educational level^c^			
Low	33 (4%)	0 (0%)	0.615
Moderate	753 (85%)	22 (88%)	..
High	99 (11%)	3 (12%)	..
Rank^d^			
Private	378 (40%)	16 (57%)	0.297
Corporal	199 (21%)	4 (14%)	..
Noncommissioned officer	245 (26%)	6 (21%)	..
Staff officer	130 (14%)	2 (7%)	..
Previous deployment(s)^e^			
Yes	417 (48%)	7 (28%)	0.053
No	460 (53%)	18 (72%)	..
Role during deployment^f^			
Inside the military base	244 (31%)	4 (31%)	0.501
Both inside and outside the military base	73 (9%)	0 (0%)	..
Outside the military base	474 (60%)	9 (69%)	..
Deployment year			
2005 or 2006	237 (25%)	24 (55%)	**<0.0001**
2007 or 2008	726 (75%)	20 (46%)	..
New deployment(s)^g^			
Yes	318 (48%)	..	..
No	344 (52%)	..	..
DES (deployment stressors) total score^h^	4.51 (3.22)	4.50 (4.95)	0.996
DDRI-F (unit social support) total score^i^	45.39 (10.19)	..	..
DDRI-L (support after deployment) total score^j^	60.43 (9.16)	..	..
ETISR-SF (early trauma) total score^k^	3.45 (3.04)	6.14 (3.32)	**0.001**

Data are *n* (%) or mean (SD). Differences in descriptive characteristics between participants with SRIP and participants without SRIP were tested with a *t* test (continuous) or *χ* ^2^ (categorical). Bold indicates significant relationship (*p* < 0.05).

Abbreviations: DES, Deployment Experience Scale; ETISR-SF, Early Trauma Inventory Self Report-Short Form; SRIP, Self-Rating Inventory for Post-traumatic Stress Disorder.

^a^Sample sizes might not add up to total because of missing data in the descriptive variables; where there is missing data, the total is indicated. Totals for participants with an SRIP measurement: ^b^ *n* = 961, ^c^ *n* = 885, ^d^ *n* = 952, ^e^ *n* = 877, ^f^ *n* = 791, ^g^ *n* = 662, ^h^ *n* = 705, ^i^ *n* = 335, ^j^ *n* = 334, ^k^ *n* = 874; totals for participants without an SRIP measurement: ^b^ *n* = 39, ^c^ *n* = 25, ^d^ *n* = 28, ^e^ *n* = 25, ^f^ *n* = 13, ^g^ *n* = 0, ^h^ *n* = 2, ^i^ *n* = 0, ^j^ *n* = 0, ^k^ *n* = 14.

### PTSD symptom increase and covariates

Mean PTSD symptom levels and probable PTSD rates at each time point are reported in Table 2. A full tabulation of the results for all analyses is shown in the Supplementary Material. At the 10-year follow-up measurement, 8% of the participants reported substantial PTSD symptoms, which was a significant decline compared to 5-year postdeployment (*p* < 0.0001). The mean PTSD symptom score also significantly declined at 10-year follow-up to a score of 27.35 (*p* = 0.046). The mixed model analysis with only the time points included showed a significant increase of PTSD symptoms at 10 years after deployment relative to predeployment (*β* = 0.84, 95% confidence intervals [CI] = 0.34–1.34).

**Table 2.** Dutch military personnel deployed to Afghanistan reporting post-traumatic stress symptoms at each time point.

	Total number of participants with available data	Above cutoff^a^	Mean PTSD score
Predeployment	680	27 (4.0%)	26.76 (5.03)
1 month	753	62 (8.2%)	27.62 (6.14)
6 months	737	63 (8.5%)	27.73 (7.07)
12 months	562	38 (6.8%)	27.02 (6.94)
2 years	528	29 (5.5%)	26.64 (5.90)
5 years	559	72 (12.9%)	28.30 (8.07)
10 years	598	48 (8.0%)	27.35 (7.20)

Data are *n*, *n* (%), or mean (SD).

Abbreviations: PTSD, post-traumatic stress disorder; SRIP, Self-Rating Inventory for Post-traumatic Stress Disorder.

a A PTSD score of 38 or higher on the SRIP was used as cutoff value.

The interactions of covariates with the change in PTSD symptoms 10-year postdeployment relative to predeployment are shown in Table 3. Age was significantly related to a lower increase in PTSD symptoms at 10 years after deployment (*β* = −0.07, 95% CI = −0.12 to −0.01), suggesting a higher increase in PTSD symptoms for younger military personnel and a lower increase in symptoms for older military personnel relative to predeployment. As age and rank were strongly correlated (*r* = 0.73), similar confounding effects were found for rank during deployment (*β* = −1.36, 95% CI = −2.38 to −0.35), where the lower ranking personnel (i.e., soldier and corporal ranks) had more increased PTSD symptoms compared to higher ranking personnel (i.e., noncommissioned and staff officers). Also, educational level was related to the increase in PTSD symptoms at 10 years postdeployment (*β* = −3.99, 95% CI = −7.27 to −0.71), where personnel with a low educational level had greater increase in symptoms than personnel with a high educational level.

**Table 3.** Covariates associated with an increase in PTSD symptoms 10 years after deployment relative to predeployment.

	Increase in PTSD symptoms 10 year postdeployment β (95% CI)	*p*-value
Age	**−0.07 (−0.12 to −0.01)**	**0.016**
Educational level		
Low	0	
Moderate	−2.48 (−5.47 to 0.52)	0.105
High	**−3.99 (−7.27 to −0.71)**	0.017
Rank		
Soldier and corporal	0	
Noncommissioned officer and staff officer	**−1.36 (−2.38 to −0.35)**	**0.009**
Previous deployment(s)		
No	0	
Yes	−0.09 (−1.12 to 0.95)	0.868
Role during deployment		
Inside	0	
Both inside and outside	**2.79 (0.79–4.79)**	0.006
Outside	1.10 (−0.08 to 2.28)	0.067
Deployment year		
2005/2006	0	
2007/2008	1.09 (−0.19 to 2.37)	0.095
New deployment(s)		
No	0	
Yes	0.45 (−0.56 to 1.47)	0.383
Deployment stressors	**0.28 (0.11–0.46)**	**0.002**
Unit social support	0.00 (−0.08 to 0.08)	0.963
Social support after deployment	**−0.12 (−0.21 to −0.03)**	**0.010**
Early general trauma	−0.06 (−0.38 to 0.25)	0.705
Early physical abuse	−0.34 (−0.78 to 0.09)	0.118
Early emotional abuse	−0.44 (−0.94 to 0.07)	0.089
Early sexual abuse	**−1.25 (−2.21 to −0.29)**	**0.011**

Bold indicates significant relationship (*p* < 0.05).

Abbreviations: CI, confidence interval; PTSD, post-traumatic stress disorder.

Reported previous sexual abuse was associated with a lower increase in PTSD symptoms at 10-year follow-up (*β* = −1.25, 95% CI = −2.21 to −0.29), whereas previous general trauma, physical abuse, and emotional abuse had no effect. Previous deployments did not have an effect on the change in PTSD symptoms. A higher level of deployment stressors was related to a greater increase in symptoms (*β* = 0.28, 95% CI = 0.11–0.46). Moreover, military personnel with a role both inside and outside the base had more increased PTSD symptoms than the group that operated only inside the base (*β* = 2.97, 95% CI = 0.79–4.79). No difference was found between personnel that operated inside the base and personnel that operated outside the base. Year of deployment was not related to the increase in PTSD symptoms, nor was the level of unit social support during deployment. Social support after deployment was associated with a lower increase in PTSD symptoms (*β* = −0.12, 95% CI = −0.21 to −0.03), suggesting a lower increase in PTSD symptoms for personnel that received more social support after return. A new deployment after the main deployment was not related to the change in PTSD symptoms.

### Trajectory analysis and associated factors

**Figure 1.**
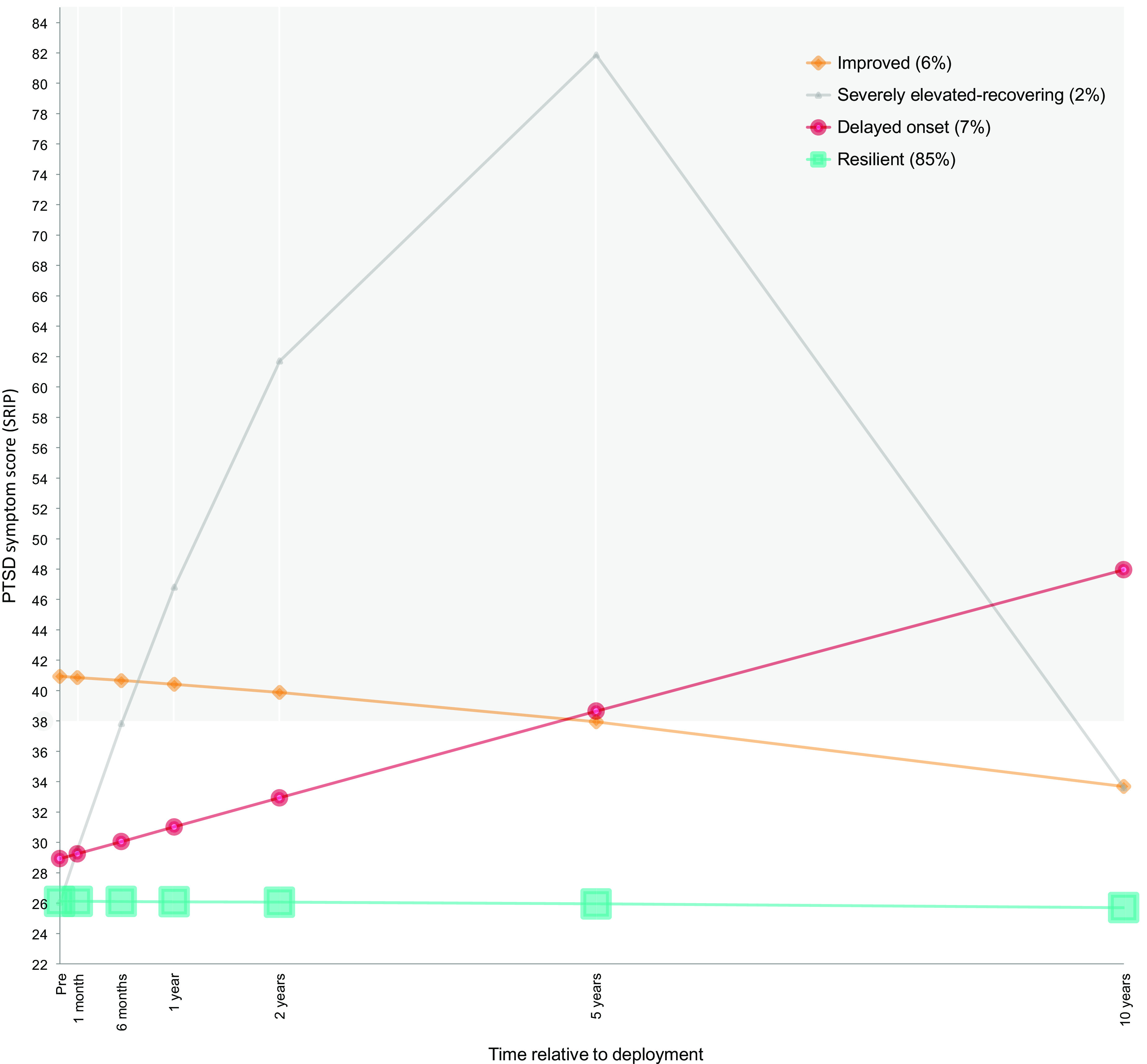
Latent developmental trajectories of post-traumatic stress symptoms. PTSD, post-traumatic stress disorder; SRIP, Self-Rating Inventory for post-traumatic stress disorder; A SRIP score of 38 was used as a cut-off to indicate substantial PTSD symptoms.

First, a series of LCGA were fitted, both with and without a quadratic term for time. The nonlinear growth curves provided better fit to the data in the majority of the models (see Supplementary Material for fit results of the models). Next, a series of GMM were conducted. The four-class GMM including a quadratic term for time produced the best solution with respect to fit and theoretical interpretation. The five-class and six-class GMMs provided better fit indices, but consisted of multiple small groups which considerably limited theoretical justification and interpretability of the identified classes. The model with four latent trajectories (see Figure 1) consisted of one large group of 822 participants (85%) with a low and stable trajectory (i.e., resilient), a smaller group of 67 participants (7%) with a trajectory of increasing symptoms reaching cut-off for PTSD between 2 and 5 years postdeployment (i.e., delayed onset), a group of 57 participants (6%) with high symptoms predeployment and shortly after deployment, but gradual recovery after 6 months postdeployment (i.e., improved), and a group of 16 participants (2%) with heavily increasing symptoms that showed recovery after 5 years postdeployment (i.e., severely elevated-recovering). Results indicated that participants in the resilient group were the least likely to reporting receiving any mental health support (24%). Of the participants in the delayed onset group, 77% received any psychological care, compared to 43% in the improved group and 80% in the severely-elevated recovered group.

We carried out multinomial logistic regression analyses to assess the associations between the assigned trajectories and different covariates (see Table 4). In comparison to the resilient group, the delayed onset group operated more often both inside and outside the military base compared to exclusively inside the base (OR = 3.22, 95% CI = 1.21–8.55), was more frequently deployed in 2007/2008 compared to 2005/2006 (OR = 2.50, 95% CI = 1.02–6.06), experienced more deployment stressors (OR = 1.15, 95% CI = 1.05–1.26), and received less social support after deployment (OR = 0.95, 95% CI = 0.91–0.99). The improved group experienced more deployment stressors (OR = 1.16, 95% CI = 1.06–1.28), less unit support during deployment (OR = 0.93, 95% CI = 0.90–0.97), less support after deployment (OR = 0.94, 95% CI = 0.91–0.98), and more physical (OR = 1.23, 95% CI = 1.01–1.51), emotional (OR = 1.64, 95% CI = 1.40–1.91), and sexual abuse (OR = 1.59, 95% CI = 1.22–2.07) during childhood compared to the resilient group. The severely elevated-recovering group was younger compared to the resilient group (OR = 0.82, 95% CI = 0.69–0.96), experienced more deployment stressors (OR = 1.26, 95% CI = 1.07–1.50), less support after deployment (OR = 0.93, 95% CI = 0.88–0.98), and more childhood sexual abuse (OR = 1.54, 95% CI = 1.04–2.27). Compared to the improved group, the delayed onset group experienced less emotional (OR = 0.75, 95% CI = 0.60–0.95) and sexual abuse (OR = 0.58, 95% CI = 0.37–0.91) during childhood. Finally, the severely elevated-recovering group was younger compared to the improved group (OR = 0.83, 95% CI = 0.70–0.98). The trajectories did not differ in rank, educational level, previous deployments, new deployments, or childhood general trauma score.

**Table 4.** Covariates associated with PTSD symptom developmental trajectories.

	Delayed onset vs. resilient	Improved vs. resilient	Severely elevated-recovering vs. resilient	Delayed onset vs. improved	Severely elevated-recovering vs. delayed onset	Severely elevated-recovering vs. improved
	OR (95% CI)	OR (95% CI)	OR (95% CI)	OR (95% CI)	OR (95% CI)	OR (95% CI)
Age	0.96 (0.91–1.01)	0.98 (0.95–1.02)	**0.82 (0.69–0.96)**	0.97 (0.92–1.03)	0.86 (0.72–1.01)	**0.83 (0.70–0.98)**
Educational level						
Moderate vs. low	0.60 (0.18–2.05)	0.58 (0.17–1.99)	^a^	1.04 (0.22–5.00)	^a^	^a^
Moderate vs. high	^a^	0.93 (0.40–2.16)	1.55 (0.20–12.1)	^a^	^a^	1.67 (0.18–15.1)
High vs. low	^a^	0.62 (0.15–2.58)	^a^	^a^	^a^	^a^
Rank						
Soldier and corporal	1.00	1.00	1.00	1.00	1.00	1.00
Noncommissioned officer and staff officer	0.58 (0.31–1.10)	1.10 (0.63–1.92)	0.06 (0.01–2.35)	0.53 (0.23–1.21)	0.10 (0.01–4.25)	0.06 (0.01–2.24)
Previous deployment(s)						
No	1.00	1.00	1.00	1.00	1.00	1.00
Yes	0.72 (0.39–1.32)	0.88 (0.50–1.57)	0.31 (0.08–1.20)	0.81 (0.36–1.85)	0.43 (0.10–1.85)	0.35 (0.08–1.51)
Role during deployment						
Both vs. inside	**3.22 (1.21–8.55)**	1.78 (0.69–4.64)	^a^	1.80 (0.50–6.50)	^a^	^a^
Both vs. outside	1.84 (0.81–4.16)	1.90 (0.81–4.47)	^a^	0.97 (0.33–2.85)	^a^	^a^
Outside vs. inside	1.76 (0.83–3.72)	0.93 (0·48–1.79)	^a^	1.90 (0.70–5.12)	^a^	^a^
Deployment year						
2005/2006	1.00	1.00	1.00	1.00	1.00	1.00
2007/2008	**2.50 (1.02–6.06)**	0.99 (0.54–1.86)	5.71 (0.46–71.2)	2.50 (0.86–7.28)	2.29 (0.16–33.2)	5.73 (0.42–77.4)
New deployment(s)						
No	1.00	1.00	1.00	1.00	1.00	1.00
Yes	0.76 (0.40–1.44)	0.68 (0.36–1.31)	1.36 (0.36–5.03)	1.11 (0.47–2.64)	1.79 (0.45–7.14)	1.99 (0.47–8.34)
Deployment stressors	**1.15 (1.05–1.26)**	**1.16 (1.06–1.28)**	**1.26 (1.07–1.50)**	0.99 (0.87–1.13)	1.10 (0.91–1.32)	1.09 (0.90–1.32)
Unit social support	0.97 (0.94–1.01)	**0.93 (0.90–0.97)**	0.94 (0.88–1.00)	1.04 (0.99–1.09)	0.97 (0.90–1.04)	1.01 (0.94–1.07)
Social support after deployment	**0.95 (0.91–0.99)**	**0.94 (0.91–0.98)**	**0.93 (0.88–0.98)**	1.01 (0.97–1.04)	0.98 (0.93–1.03)	0.98 (0.94–1.03)
Early general trauma	1.04 (0.93–1.15)	1.06 (0.93–1.21)	1.01 (0.77–1.33)	0.98 (0.92–1.04)	0.98 (0.77–1.24)	0.95 (0.76–1.20)
Early physical abuse	1.10 (0.88–1.38)	**1.23 (1.01–1.51)**	0.80 (0.34–1.89)	0.89 (0.66–1.20)	0.73 (0.30–1.78)	0.65 (0.26–1.59)
Early emotional abuse	1.23 (0.99–1.53)	**1.64 (1.40–1.91)**	1.01 (0.54–1.91)	**0.75 (0.60–0.95)**	0.82 (0.42–1.60)	0.62 (0.32–1.18)
Early sexual abuse	0.92 (0.60–1.41)	**1.59 (1.22–2.07)**	**1.54 (1.04–2.27)**	**0.58 (0.37–0.91)**	1.68 (0.96–2.91)	0.97 (0.67–1.39)

Bold indicates significant relationship (*p* < 0.05).

Abbreviations: CI, confidence interval; OR, odds ratio.

a Analysis could not be performed due to too small group sizes in the categorical covariates and/or trajectories.

## Discussion

In the current study, we assessed the effect of deployment on post-traumatic stress symptoms 10 years postdeployment in a large sample of Dutch Afghanistan veterans that were deployed as part of the ISAF mission. During the mission, service members experienced high-intensity war-zone stressors such as exposure to enemy fire, armed combat, and seeing seriously injured colleagues and civilians [17]. Ten years after returning home, the average level of PTSD symptoms was still increased compared to the predeployment level. However, the probable 10-year PTSD prevalence of 8% and the average PTSD symptom score of 27.4 were significantly declined compared to 5-year postdeployment (respectively 12.9% and 28.3). As hypothesized, this indicates that the previously identified, subsequent increase in PTSD symptoms 5 years after deployment [6] tapers off in the following years. Our study also showed that previously identified risk factors like younger age, lower rank, more deployment stressors, and less social support are still relevant 10 years after deployment. As a combination of duties both inside and outside the military base was exclusively related to the increase in PTSD symptoms at 10-year, personnel with a combined role during deployment might be a well-defined group that could benefit from long-term monitoring to prevent worsening of symptoms between 5 and 10 years postdeployment. To our surprise, our results suggest that previous sexual abuse is associated with a lower increase in PTSD symptoms at 10-year follow-up. Paradoxically, in the literature, early sexual abuse is reported as a risk factor for developing PTSD after experiencing traumatic events in adulthood [21,22].

Using seven measurements beginning 1-month predeployment through 10 years postdeployment, we found four different trajectories of PTSD symptom development. The largest majority (85%) of deployed military personnel did not develop PTSD symptoms in the 10 years after returning home. This percentage falls into the range of identified resilient trajectory group size in similar military cohorts (range: 76–90%) [4,7–10,23,24], and supports the idea that most service members deployed to war zones show enduring resiliency despite exposure to traumatic stressors. This study provides an addition to this literature by showing that their resiliency is sustained over a long period after deployment. However, a considerable group (15%) showed symptomatic courses. Our findings regarding the number and shape of these symptomatic trajectories are comparable with several other studies, although the majority of these studies had shorter follow-up periods. Of note is the study by Porter et al. [10] using data from a mixed sample of U.S. active duty and separated military personnel of the Millennium Cohort Study with a follow-up period of 9 years. The improved trajectory (6%) has been identified in other military populations, with comparable membership rates (5%) among U.S. military service members [10], but slightly lower rates (4%) among U.K. armed force members [7] and higher rates (9%) among U.S. military personnel [8] deployed to Afghanistan and Iraq. The severely elevated-recovering trajectory (2%) is compatible with the elevated-recovering trajectory (3%) identified by Porter et al. [10] in a sample of U.S. military personnel, although their reported elevation in symptoms was not as high as in our sample. The delayed onset trajectory (7%) was also identified with a somewhat lower membership rate (5%) in the U.S. military sample by Porter et al. [10], and is consistent with prior work showing that symptoms often increase after a temporal lag relative to the exposure to a traumatic event [25]. In our previous 5-year follow-up report on the PRISMO cohort [6], we identified a resilient, recovered, and delayed onset PTSD trajectory. The four-class solution in the present 10-year follow-up probably resulted from the seperation of a small group of individuals who showed major recovery between 5 and 10 years postdeployment from the original delayed onset trajectory.

The reported decline in probable PTSD prevalence from 13% (5 years postdeployment) to 8% (10 years postdeployment) is reflected in the dynamics of the identified developmental trajectories. Obviously, the most striking drop in symptom score between 5 and 10 years after deployment is demonstrated by the severely elevated-recovering group. Also, the improved group shows a substantial decline from probable PTSD 5 years after deployment to a mean score clearly beneath cut-off 10 years after deployment. This decrease in PTSD symptom level could be the result of successful treatment, or might reflect the natural course of the disorder. Interestingly, the delayed onset group shows increasing symptom levels between 5 and 10 years postdeployment. Healthcare professionals should be aware of this group of veterans with increasing treatment demands up to at least 10 years postdeployment, despite an average decline in symptoms of the population as a whole. Individuals belonging to the delayed onset class might in fact be a subpopulation of PTSD patients, with possibly different psychological and neurobiological underpinnings, for which targeted early interventions might be beneficial to prevent worsening of PTSD symptoms later in life. The difficulty remains, however, how veterans with an increased risk for delayed onset PTSD can be identified in an early stadium where symptoms are still subclinical or even minimally present.

Our covariate analysis demonstrated that veterans in the delayed onset trajectory experienced a higher threat level during deployment and perceived less social support after returning home compared to veterans in the resilient group. However, this also applied for the other symptomatic trajectories. Unfortunately, no differences in variables included in the present study were found between individuals in the delayed onset group and the severely elevated-recovering group. It is important to clarify why veterans in the severely elevated-recovering trajectory are able to show a striking drop in PTSD symptoms between 5 and 10 years after deployment, while the delayed onset group shows increasing symptom levels after 5 years. Differences in treatment utilization might explain this inconsistency in symptom reduction. To our surprise, our results showed that participants in the delayed onset group reported high use of mental health support (77%), similar to the severely elevated-recovering group (80%). Additional research is therefore needed to elucidate why veterans in the delayed onset group do not seem to benefit as much as the severely elevated-recovering group after seeking help, and should focus on received treatment type, timing, and outcome. Recently identified biological mechanisms in successful treatment of PTSD like DNA methylation reversal [26] and the role of underlying moral injury in treatment effectivity [27] are also of large interest and may offer new perspectives. In addition, continued effort should be put in the identification and addressment of current PTSD symptoms, as 23% of the veterans in the delayed onset group did not receive any psychological help.

Several limitations of the current study should be mentioned. First, the use of self-report measures to obtain PTSD symptom levels as a proxy for clinical diagnoses is imperfect. Although standardized and validated screening instruments were used, it might have resulted in higher prevalence estimates compared with clinician-administered interviews [28,29]. However, its use remained consistent across time points. In addition, the reported PTSD symptoms are not necessarily the result of traumatic events during deployment. Even though we were able to maintain approximately 60% of the original sample at 10-year follow-up, the influence of nonresponse on the study findings cannot be ruled out. Another important limitation is the small group size of the “severely elevated-recovering” trajectory, which contained only 2% of our sample. The mean PTSD symptom score of this trajectory at 5 years postdeployment was near the maximum of the scale, and the variance of PTSD symptom scores in the full sample at 5 years was large compared to the other time points. The “severely elevated-recovering” trajectory might therefore be solely defined by this individual data point. Although the four-class model including this trajectory over performed the three-class model, one should be extra careful when drawing conclusions from this trajectory. Finally, the absence of information on received treatment type and timing, incurred traumatic brain injury or other types of physical injury during deployment, pre-existing psychiatric disorders, and comorbidity with other psychiatric diagnoses is a limitation of the present study. The results of this study, however, also address limitations of previous research in several ways. The predeployment measurement allowed evaluation of PTSD symptom trajectories beginning prior to deployment. We were therefore able to reveal elevated symptom levels before deployment in the improved trajectory, which would otherwise remain unobserved. Furthermore, this study has a large number of follow-up measurements during a long period of time, which enables the examination of smaller fluctuations in PTSD symptoms and the differentiation between trajectories up to 10 years after deployment.

Overall, we found a probable PTSD prevalence of 8% in a sample of Afghanistan veterans 10 years after their deployment. This implicates that the long-term symptom increase measured at 5 years postdeployment decreased partly in the following years. Of note is the delayed onset group that experienced increasing symptom levels between 5 and 10 years postdeployment, and does not seem to be able to show significant symptom reduction after seeking mental health support. These findings raise critical questions about the origin of this inconsistency in symptom reduction. Future research is therefore needed to elucidate which factors may contribute to the worsening of PTSD symptoms and probable treatment resistance in the delayed onset trajectory in order to develop and implement alternative treatment strategies for this group of veterans.

## Data Availability

The data that support the findings of this study are available from the corresponding author, S.v.d.W., upon reasonable request.

## Supplementary material

For supplementary material accompanying this paper visit https://doi.org/10.1192/j.eurpsy.2020.113. click here to view supplementary material
