# Supplementary material for: Long-term development of post-traumatic stress symptoms and associated risk factors in military service members deployed to Afghanistan: Results from the PRISMO 10-year follow-up
Source: Eur Psychiatry. 2020 Dec 21;64(1):e10. doi: 10.1192/j.eurpsy.2020.113 (PMC8057418; doi:10.1192/j.eurpsy.2020.113)
Supplement: Supplementary file 1 [file S0924933820001133sup001.doc]

Supplementary material

**Covariates**

Data on education was categorized into three levels of education: low (some years of high school), medium (finished high school), and high (college or university education). Rank was divided in four categories: private, corporal, non-commissioned officer, and staff officer. Previous deployments were dichotomized (yes or no). The participant’s role during the mission was stratified into ‘inside the military base’, ‘both inside and outside the military base’ and ‘outside the military base’.

**Mental health support**

Received psychological care was assessed by the item “Have you ever received any care for psychological health complaints after your deployment?”. We defined psychological care as any received care for psychological health complaints provided by a GP, social worker, psychologist, psychiatrist, or other mental health specialist. Due to the high percentage of missing values (Table S1), the psychological care variable was not imputed and therefore not tested as an associated factor for PTSD trajectories in the adjusted multinomial regression models.

***Table S1:* Participants reporting receiving any psychological care, separated for PTSD**

**trajectory.**

|  | **Participants that received any psychological care** | **Missing data in outcome variable** |
| --- | --- | --- |
| All (n=963) | 28.5% | 35.9% |
| Resilient (n=845) | 24.1% | 35.3% |
| Delayed onset (n=50) | 77.1% | 30.0% |
| Improved (n=55) | 43.3% | 45.5% |
| Severely-elevated recovering (n=13) | 80.0% | 61.5% |

**Missing data analyses**

The association of study drop-out with symptom levels was studied by correlating the occurrence of missing values to the posttraumatic stress symptom scores on the previous time point. The symptom level at six months after deployment was related to the occurrence of missing values at two years (r=0.113; p=0.002), five years (r=0.080; p=0.029), and ten years (r=0.086; p=0.020) after deployment. The symptom levels at one year and five years after deployment were related to the occurrence of missing values at ten years after deployment (respectively r=0.114; p=0.007 and r=0.091; p=0.031). The missing data patterns are presented in Table S2. Considering that in mixed model analysis the missing data are not assumed to be missing completely at random, and that the results obtained from a mixed model analysis with multiple imputation can be quite unstable [30], no multiple imputation techniques were used prior to the mixed model analyses. For the latent trajectory growth mixture model, missing values on the potential modifying factors were handled with multiple imputation prior the analyses.

***Table S2:* Missing data patterns for the posttraumatic stress symptom scores at each time point.**

| **Time points** | | | | | | | |
| --- | --- | --- | --- | --- | --- | --- | --- |
| **Pre-deployment** | **1 month** | **6 months** | **1 year** | **2 years** | **5 years** | **10 years** | **n^a^** |
|  |  |  |  |  |  |  | 223 |
| X |  |  |  |  |  |  | 53 |
| X | X |  |  |  |  |  | 23 |
|  | X |  |  |  |  |  | 18 |
|  |  |  | X |  |  |  | 13 |
|  |  |  |  |  |  | X | 30 |
|  |  |  |  | X |  |  | 23 |
|  |  |  | X | X |  |  | 16 |
|  |  |  |  |  | X |  | 15 |
|  |  |  |  | X | X |  | 16 |
|  |  |  |  | X | X | X | 14 |
|  |  |  | X | X | X |  | 19 |
|  |  |  | X | X | X | X | 57 |
|  |  | X | X | X | X | X | 36 |
| X |  | X | X | X | X | X | 18 |
| X |  |  | X | X | X | X | 27 |
|  | X | X | X | X | X | X | 37 |
| X | X | X | X | X | X | X | 44 |

*Note:* X indicates a missing value for that time point; ^a^n indicates the number of participants that had the corresponding missing data pattern; patterns that occurred in less than 1% of the participants were omitted.

**Multiple imputation procedure**

Multiple imputation was performed in SPSS version 25 using multivariate imputation by chained equations (MICE) with predictive mean matching. A total of 10 imputations were used. The imputation model included the Early Trauma Inventory Self Report-Short Form (ETISR-SF), the Self-Rated Inventory for Post-traumatic Stress Disorder (SRIP), the Deployment Experience Scale (DES), the Deployment Risk and Resilience Inventory-1(DRRI) part F and L, and the baseline descriptive variables (i.e. age, gender, education level, rank, previous deployments, function during deployment, deployment year, and new deployments). The missing items in the ETISR-SF, DES, and DRRI were imputed at the item level. Convergence plots were used to check if the imputed values had the expected variation between the iterations. The total scores of the questionnaires were calculated by summing the imputed item scores.

**Mixed model analyses**

***Table S3:* Parameter estimates for change in posttraumatic stress symptoms over time relative to pre-deployment status without interactions with potential covariatescovariate** (n=963).

|  | **Time-effect** | |
| --- | --- | --- |
|  | Coefficient (95% CI) | P-value |
| Intercept (pre-deployment) | 26.72 (26.36 – 27.09) | <0.0001 |
| Δ 1 month^a^ | 0.92 (0.46 – 1.38) | <0.0001 |
| Δ 6 months^a^ | 0.99 (0.52 – 1.45) | <0.0001 |
| Δ 1 year^a^ | 0.38 (-0.14 – 0.90) | 0.148 |
| Δ 2 years^a^ | 0.14 (-0.39 – 0.67) | 0.597 |
| Δ 5 years^a^ | 1.62 (1.11 – 2.14) | <0.0001 |
| Δ 10 years^a^ | 0.84 (0.34 – 1.34) | 0.001 |

Note: ^a^ Δ indicates the difference relative to pre-deployment status; 95% CI = 95%

Confidence Interval.

***Table S4:* Parameter estimates for change in posttraumatic stress symptoms over time relative to pre-deployment status with age as covariate** (n=963).

|  | **Effect** | | **Interaction time x age** | |
| --- | --- | --- | --- | --- |
|  | Coefficient (95% CI) | P-value | Coefficient (95% CI) | P-value |
| Intercept (pre-deployment) | 27.83 (26.58 – 29.07) | <0.0001 |  |  |
| Δ 1 month^a^ | 2.09 (0.52 – 3.65) | 0.009 | -0.04 (-0.09 – 0.01) | 0.126 |
| Δ 6 months^a^ | 2.15 (0.59 – 3.71) | 0.007 | -0.04 (-0.09 – 0.01) | 0.124 |
| Δ 1 year^a^ | 2.08 (0.35 – 3.82) | 0.018 | -0.06 (-0.11 – -0.01) | 0.044 |
| Δ 2 years^a^ | 0.27 (-1.53 – 2.08) | 0.767 | -0.01(-0.06 – 0.05) | 0.846 |
| Δ 5 years^a^ | 4.64 (2.88 – 6.40) | <0.0001 | -0.10 (-0.15 – -0.04) | 0.001 |
| Δ 10 years^a^ | 2.84 (1.14 – 4.53) | 0.001 | -0.07 (-0.12 – -0.01) | 0.016 |
| Age | -0.04 (-0.08 – 0.01) | 0.076 |  |  |

Note: ^a^ Δ indicates the difference relative to pre-deployment status; 95% CI = 95% Confidence Interval.

***Table S5:* Parameter estimates for change in posttraumatic stress symptoms over time relative to pre-deployment status with educational level as covariate** (n=963).

|  | **Effect** | | **Interaction time x education_medium_^b^** | | **Interaction time x education_high_^b^** | |
| --- | --- | --- | --- | --- | --- | --- |
|  | Coefficient (95% CI) | P-value | Coefficient (95% CI) | P-value | Coefficient (95%CI) | P-value |
| Intercept (pre-deployment) | 28.04 (26.09 – 29.99) | <0.0001 |  |  |  |  |
| Δ 1 month^a^ | 0.95 (-1.43 – 3.32) | 0.434 | -0.07 (-2.49 – 2.36) | 0.955 | -0.44 (-3.21 – 2.33) | 0.755 |
| Δ 6 months^a^ | 1.10 (-1.33 – 3.53) | 0.374 | -0.15 (-2.63 – 2.33) | 0.903 | -0.78 (-3.58 – 2.02) | 0.584 |
| Δ 1 year^a^ | 0.29 (-2.88 – 3.47) | 0.857 | 0.07 (-3.16 – 3.29) | 0.968 | -0.54 (-4.03 – 2.93) | 0.758 |
| Δ 2 years^a^ | -2.29 (-5.89 – 1.32) | 0.214 | 2.50 (-1.15 – 6.16) | 0.179 | 2.17 (-1.69 – 6.04) | 0.270 |
| Δ 5 years^a^ | -1.00 (-4.62 – 2.61) | 0.587 | 2.61 (-1.05 – 6.27) | 0.162 | 2.01 (-1.88 – 5.89) | 0.311 |
| Δ 10 years^a^ | 3.34 (0.40 – 6.28) | 0.026 | -2.48 (-5.47 – 0.52) | 0.105 | -3.99 (-7.27 – -0.71) | 0.017 |
| Education_medium_^b^ | -1.30 (-3.29 – 0.69) | 0.092 |  |  |  |  |
| Education_high_^b^ | -1.95 (-4.21 – 0.32) | 0.201 |  |  |  |  |

Note: ^a^ Δ indicates the difference relative to pre-deployment status; ^b^ reference category is the group with a low educational level; 95% CI = 95% Confidence Interval; Education_medium_ = medium educational level; Education_high_ = high educational level.

***Table S6:* Parameter estimates for change in posttraumatic stress symptoms over time relative to pre-deployment status with military rank as covariate** (n=963).

|  | **Effect** | | **Interaction time x rank^b^** | |
| --- | --- | --- | --- | --- |
|  | Coefficient (95% CI) | P-value | Coefficient (95% CI) | P-value |
| Intercept (pre-deployment) | 26.90 (26.44 – 27.37) | <0.0001 |  |  |
| Δ 1 month^a^ | 1.07 (0.48 – 1.66) | <0.0001 | -0.39 (-1.33 – 0.55) | 0.413 |
| Δ 6 months^a^ | 1.22 (0.61 – 1.83) | <0.0001 | -0.59 (-1.53 – 0.36) | 0.224 |
| Δ 1 year^a^ | 0.62 (-0.10 – 1.34) | 0.091 | -0.54 (-1.58 – 0.50) | 0.308 |
| Δ 2 years^a^ | 0.17 (-0.60 – 0.93) | 0.668 | -0.08 (-1.15 – 0.98) | 0.879 |
| Δ 5 years^a^ | 2.54 (1.81 – 3.26) | <0.0001 | -1.81 (-2.85 – -0.77) | 0.001 |
| Δ 10 years^a^ | 1.48 (0.80 – 2.17) | <0.0001 | -1.36 (-2.38 – -0.35) | 0.009 |
| Rank^b^ | -0.44 (-1.19 – 0.31) | 0.253 |  |  |

Note: ^a^ Δ indicates the difference relative to pre-deployment status; ^b^ the rank parameter indicates the differences between non-commissioned officer and staff officer ranks versus soldier and corporal ranks (reference category); 95% CI = 95% Confidence Interval.

***Table S7:* Parameter estimates for change in posttraumatic stress symptoms over time relative to pre-deployment status with previous deployment(s) as covariate** (n=963).

|  | **Effect** | | **Interaction time x previous deployments^b^** | |
| --- | --- | --- | --- | --- |
|  | Coefficient (95% CI) | P-value | Coefficient (95% CI) | P-value |
| Intercept (pre-deployment) | 26.84 (26.33 – 27.35) | <0.0001 |  |  |
| Δ 1 month^a^ | 0.90 (0.25 – 1.56) | 0.007 | 0.00 (-0.94 – 0.94) | 0.999 |
| Δ 6 months^a^ | 1.14 (0.47 – 1.81) | 0.001 | -0.41 (-1.36 – 0.54) | 0.398 |
| Δ 1 year^a^ | 0.75 (-0.01 – 1.51) | 0.055 | -0.71 (-1.76 – 0.35) | 0.188 |
| Δ 2 years^a^ | 0.05 (-0.75 – 0.85) | 0.905 | 0.22 (-0.87 – 1.31) | 0.695 |
| Δ 5 years^a^ | 1.73 (0.96 – 2.50) | <0.0001 | -0.41 (-1.47 – 0.65) | 0.449 |
| Δ 10 years^a^ | 0.84 (0.10 – 1.57) | 0.027 | -0.09 (-1.12 – 0.95) | 0.868 |
| Previous deployments^b^ | -0.42 (-1.16 – 0.32) | 0.263 |  |  |

Note: ^a^ Δ indicates the difference relative to pre-deployment status; ^b^ reference category is the group without previous deployments; 95% CI = 95% Confidence Interval.

***Table S8:* Parameter estimates for change in posttraumatic stress symptoms over time relative to pre-deployment status with role during deployment as covariate** (n=963).

|  | **Effect** | | **Interaction time x role_both_^b^** | | **Interaction time x role_outside_^b^** | |
| --- | --- | --- | --- | --- | --- | --- |
|  | Coefficient (95% CI) | P-value | Coefficient (95% CI) | P-value | Coefficient (95%CI) | P-value |
| Intercept (pre-deployment) | 26.61 (25.90 – 27.31) | <0.0001 |  |  |  |  |
| Δ 1 month^a^ | 0.53 (-0.36 – 1.42) | 0.241 | 0.77 (-1.03 – 2.56) | 0.403 | 0.61 (-0.48 – 1.70) | 0.274 |
| Δ 6 months^a^ | 0.40 (-0.46 – 1.27) | 0.362 | 0.86 (-0.91 – 2.64) | 0.340 | 0.97 (-0.13 – 2.06) | 0.083 |
| Δ 1 year^a^ | -0.36 (-1.27 – 0.55) | 0.438 | -0.04 (-1.97 – 1.89) | 0.967 | 1.19 (0.00 – 2.38) | 0.049 |
| Δ 2 years^a^ | -0.16 (-1.07 – 0.75) | 0.732 | -0.38 (-2.51 – 1.75) | 0.725 | 0.57 (-0.66 – 1.80) | 0.362 |
| Δ 5 years^a^ | 0.70 (-0.21 – 1.61) | 0.133 | 1.42 (-0.63 – 3.47) | 0.175 | 1.86 (0.66 – 3.06) | 0.002 |
| Δ 10 years^a^ | 0.18 (-0.72 – 1.09 ) | 0.691 | 2.79 (0.79 – 4.79) | 0.006 | 1.10 (-0.08 – 2.28) | 0.067 |
| Role_both_^b^ | 0.35 (-0.52 – 1.21) | 0.434 |  |  |  |  |
| Role_outside_^b^ | 0.72 (-0.73 – 2.17) | 0.331 |  |  |  |  |

Note: ^a^ Δ indicates the difference relative to pre-deployment status; ^b^ reference category is the group with a role inside the military base; 95% CI = 95% Confidence Interval; Role_both_ = role both inside and outside the military base; Role_outside_ = role outside the military base.

***Table S9:* Parameter estimates for change in posttraumatic stress symptoms over time relative to pre-deployment status with deployment year as covariate** (n=963).

|  | **Effect** | | **Interaction time x year_2007/2008_^b^** | |
| --- | --- | --- | --- | --- |
|  | Coefficient (95% CI) | P-value | Coefficient (95% CI) | P-value |
| Intercept (pre-deployment) | 25.12 (23.26 – 26.99) | <0.0001 |  |  |
| Δ 1 month^a^ | -0.05 (-2.47 – 2.36) | 0.965 | 0.54 (-0.75 – 1.82) | 0.413 |
| Δ 6 months^a^ | 0.82 (-1.48 – 3.13) | 0.484 | 0.10 (-1.14 – 1.33) | 0.879 |
| Δ 1 year^a^ | 2.07 (-0.43 – 4.56) | 0.104 | -0.94 (-2.28 – 0.40) | 0.169 |
| Δ 2 years^a^ | 1.20 (-1.19 – 3.59) | 0.324 | -0.61 (-1.91 – 0.70) | 0.362 |
| Δ 5 years^a^ | -1.05 (-3.43 – 1.32) | 0.385 | 1.55 (0.26 – 2.84) | 0.019 |
| Δ 10 years^a^ | -1.05 (-3.42 – 1.31) | 0.382 | 1.09 (-0.19 – 2.37) | 0.095 |
| Year_2007/2008_^b^ | 0.91 (-0.09 – 1.90) | 0.074 |  |  |

Note: ^a^ Δ indicates the difference relative to pre-deployment status; ^b^ reference category is the group deployed in 2005 and 2006; 95% CI = 95% Confidence Interval.

***Table S10:* Parameter estimates for change in posttraumatic stress symptoms over time relative to pre-deployment status with new deployment(s) as covariate** (n=963).

|  | **Effect** | | **Interaction time x new deployments^b^** | |
| --- | --- | --- | --- | --- |
|  | Coefficient (95% CI) | P-value | Coefficient (95% CI) | P-value |
| Intercept (pre-deployment) | 27.43 (26.83 – 28.02) | <0.0001 |  |  |
| Δ 1 month^a^ | 0.49 (-0.25 – 1.22) | 0.189 | 0.11 (-0.95 – 1.17) | 0.841 |
| Δ 6 months^a^ | 0.20 (-0.52 – 0.92) | 0.588 | 0.84 (-0.20 – 1.89) | 0.113 |
| Δ 1 year^a^ | -0.26 (-1.02 – 0.51) | 0.512 | 0.45 (-0.65 – 1.55) | 0.422 |
| Δ 2 years^a^ | -0.55 (-1.31 – 0.22) | 0.162 | 0.71 (-0.42 – 1.83) | 0.218 |
| Δ 5 years^a^ | 1.59 (0.83 – 2.36) | <0.0001 | -0.59 (-1.67 – 0.50) | 0.288 |
| Δ 10 years^a^ | 0.41 (-0.29 – 1.11) | 0.254 | 0.45 (-0.56 – 1.47) | 0.383 |
| New deployments^b^ | -1.34 (-2.18 – -0.49) | 0.002 |  |  |

Note: ^a^ Δ indicates the difference relative to pre-deployment status; ^b^ reference category is the group without new deployments; 95% CI = 95% Confidence Interval.

***Table S11:* Parameter estimates for change in posttraumatic stress symptoms over time relative to pre-deployment status with deployment experience total score as covariate** (n=963).

|  | **Effect** | | **Interaction time x DES** | |
| --- | --- | --- | --- | --- |
|  | Coefficient (95% CI) | P-value | Coefficient (95% CI) | P-value |
| Intercept (pre-deployment) | 25.71 (24.97 – 26.44) | <0.0001 |  |  |
| Δ 1 month^a^ | -0.37 (-1.22 – 0.48) | 0.394 | 0.25 (0.10 – 0.40) | 0.001 |
| Δ 6 months^a^ | -0.55 (-1.44 – 0.35) | 0.229 | 0.27 (0.11 – 0.43) | 0.001 |
| Δ 1 year^a^ | -1.26 (-2.24 – -0.29) | 0.011 | 0.33 (0.15 – 0.51) | <0.0001 |
| Δ 2 years^a^ | -0.61 (-1.61 – 0.39) | 0.230 | 0.09 (-0.09 – 0.28) | 0.317 |
| Δ 5 years^a^ | 0.38 (-0.60 – 1.36) | 0.451 | 0.27 (0.09 – 0.45) | 0.003 |
| Δ 10 years^a^ | -0.39 (-1.34 – 0.56) | 0.426 | 0.28 (0.11 – 0.46) | 0.002 |
| DES total score | 0.24 (0.11 – 0.37) | <0.0001 |  |  |

Note: ^a^ Δ indicates the difference relative to pre-deployment status; DES = Deployment Experience Scale; 95% CI = 95% Confidence Interval.

***Table S12:* Parameter estimates for change in posttraumatic stress symptoms over time relative to pre-deployment status with unit social support as covariate** (n=963).

|  | **Effect** | | **Interaction time x DDRI-F** | |
| --- | --- | --- | --- | --- |
|  | Coefficient (95% CI) | P-value | Coefficient (95% CI) | P-value |
| Intercept (pre-deployment) | 32.52 (29.75 – 35.28) | <0.0001 |  |  |
| Δ 1 month^a^ | 1.05 (-2.28 – 4.39) | 0.535 | 0.00 (-0.07 – 0.07) | 0.932 |
| Δ 6 months^a^ | 4.73 (1.35 – 8.11) | 0.006 | -0.09 (-0.16 – -0.01) | 0.020 |
| Δ 1 year^a^ | -0.71 (-3.99 – 2.57) | 0.670 | 0.01 (-0.06 – 0.08) | 0.771 |
| Δ 2 years^a^ | -1.60 (-5.27 – 2.07) | 0.392 | 0.02 (-0.06 – 0.10) | 0.575 |
| Δ 5 years^a^ | 1.38 (-2.24 – 5.01) | 0.455 | 0.00 (-0.07 – 0.08) | 0.923 |
| Δ 10 years^a^ | 0.95 (-2.80 – 4.70) | 0.621 | 0.00 (-0.08 – 0.08) | 0.963 |
| DDRI-F total score | -0.12 (-0.18 – -0.06) | <0.0001 |  |  |

Note: ^a^ Δ indicates the difference relative to pre-deployment status; DDRI-F = Deployment Risk and Resilience Inventory-1 Section F; 95% CI = 95% Confidence Interval.

***Table S13:* Parameter estimates for change in posttraumatic stress symptoms over time relative to pre-deployment status with social support after deployment as covariate** (n=963).

|  | **Effect** | | **Interaction time x DDRI-L** | |
| --- | --- | --- | --- | --- |
|  | Coefficient (95% CI) | P-value | Coefficient (95% CI) | P-value |
| Intercept (pre-deployment) | 37.85 (33.94 – 41.76) | <0.0001 |  |  |
| Δ 1 month^a^ | 2.21 (-2.64 – 7.07) | 0.371 | -0.02 (-0.10 – 0.06) | 0.645 |
| Δ 6 months^a^ | 10.79 (6.00 – 15.58) | <0.0001 | -0.16 (-0.24 – -0.08) | <0.0001 |
| Δ 1 year^a^ | 6.99 (2.11 – 11.88) | 0.005 | -0.12 (-0.20 – -0.04) | 0.004 |
| Δ 2 years^a^ | 3.35 (-1.79 – 8.49) | 0.201 | -0.06 (-0.15 – 0.02) | 0.147 |
| Δ 5 years^a^ | 10.95 (5.60 – 16.30) | <0.0001 | -0.15 (-0.24 – -0.06) | 0.001 |
| Δ 10 years^a^ | 8.00 (2.56 – 13.44) | 0.004 | -0.12 (-0.21 – -0.03) | 0.010 |
| DDRI-L total score | -0.18 (-0.24 – -0.12) | <0.0001 |  |  |

Note: ^a^ Δ indicates the difference relative to pre-deployment status; DDRI-L = Deployment Risk and Resilience Inventory-1 Section L; 95% CI = 95% Confidence Interval.

***Table S14:* Parameter estimates for change in posttraumatic stress symptoms over time relative to pre-deployment status with early general trauma score as covariate** (n=963).

|  | **Effect** | | **Interaction time x ETISR-SF general trauma** | |
| --- | --- | --- | --- | --- |
|  | Coefficient (95% CI) | P-value | Coefficient (95% CI) | P-value |
| Intercept (pre-deployment) | 25.90 (25.32 – 26.48) | <0.0001 |  |  |
| Δ 1 month^a^ | 1.03 (0.30 – 1.76) | 0.006 | -0.06 (-0.35 – 0.22) | 0.670 |
| Δ 6 months^a^ | 0.45 (-0.29 – 1.20) | 0.233 | 0.26 (-0.03 – 0.55) | 0.083 |
| Δ 1 year^a^ | 0.68 (-0.13 – 1.50) | 0.100 | -0.16 (-0.48 – 0.16) | 0.330 |
| Δ 2 years^a^ | 0.25 (-0.59 – 1.09) | 0.554 | -0.04 (-0.37 – 0.29) | 0.821 |
| Δ 5 years^a^ | 1.61 (0.80 – 2.43) | <0.0001 | 0.01 (-0.31 – 0.33) | 0.959 |
| Δ 10 years^a^ | 0.98 (0.18 – 1.78) | 0.016 | -0.06 (-0.38 – 0.25) | 0.705 |
| ETISR-SF general trauma score | 0.42 (0.19 – 1.78) | <0.0001 |  |  |

Note: ^a^ Δ indicates the difference relative to pre-deployment status; ETISR-SF = Early Trauma Inventory Self-Report-Short Form; 95% CI = 95% Confidence Interval.

***Table S15:* Parameter estimates for change in posttraumatic stress symptoms over time relative to pre-deployment status with early physical abuse score as covariate** (n=963).

|  | **Effect** | | **Interaction time x ETISR-SF physical abuse** | |
| --- | --- | --- | --- | --- |
|  | Coefficient (95% CI) | P-value | Coefficient (95% CI) | P-value |
| Intercept (pre-deployment) | 26.19 (25.74 – 26.63) | <0.0001 |  |  |
| Δ 1 month^a^ | 1.18 (0.62 – 1.74) | <0.0001 | -0.29 (-0.69 – 0.11) | 0.149 |
| Δ 6 months^a^ | 1.21 (0.64 – 1.78) | <0.0001 | -0.25 (-0.64 – 0.14) | 0.201 |
| Δ 1 year^a^ | 0.58 (-0.05 – 1.22) | 0.070 | -0.28 (-0.71 – 0.16) | 0.215 |
| Δ 2 years^a^ | 0.29 (-0.37 – 0.94) | 0.389 | -0.10 (-0.54 – 0.33) | 0.640 |
| Δ 5 years^a^ | 1.40 (0.76 – 2.04) | <0.0001 | 0.21 (-0.22 – 0.64) | 0.344 |
| Δ 10 years^a^ | 1.09 (0.48 – 1.71) | <0.0001 | -0.34 (-0.78 – 0.09) | 0.118 |
| ETISR-SF physical abuse score | 0.61 (0.30 – 0.93) | <0.0001 |  |  |

Note: ^a^ Δ indicates the difference relative to pre-deployment status; ETISR-SF = Early Trauma Inventory Self-Report-Short Form; 95% CI = 95% Confidence Interval.

***Table S16:* Parameter estimates for change in posttraumatic stress symptoms over time relative to pre-deployment status with early emotional abuse score as covariate** (n=963).

|  | **Effect** | | **Interaction time x ETISR-SF emotional abuse** | |
| --- | --- | --- | --- | --- |
|  | Coefficient (95% CI) | P-value | Coefficient (95% CI) | P-value |
| Intercept (pre-deployment) | 26.16 (25.75 – 26.56) | <0.0001 |  |  |
| Δ 1 month^a^ | 0.76 (0.25 – 1.27) | 0.004 | 0.27 (-0.19 – 0.72) | 0.253 |
| Δ 6 months^a^ | 0.98 (0.46 – 1.50) | <0.0001 | 0.03 (-0.43 – 0.50) | 0.890 |
| Δ 1 year^a^ | 0.50 (-0.08 – 1.08) | 0.089 | -0.34 (-0.84 – 0.17) | 0.192 |
| Δ 2 years^a^ | 0.03 (-0.56 – 0.63) | 0.915 | 0.25 (-0.26 – 0.76) | 0.333 |
| Δ 5 years^a^ | 1.32 (0.74 – 1.90) | <0.0001 | 0.52 (0.01 – 1.02) | 0.044 |
| Δ 10 years^a^ | 1.09 (0.53 – 1.65) | <0.0001 | -0.44 (-0.94 – 0.07) | 0.089 |
| ETISR-SF emotional abuse score | 1.32 (0.95 – 1.69) | <0.0001 |  |  |

Note: ^a^ Δ indicates the difference relative to pre-deployment status; ETISR-SF = Early Trauma Inventory Self-Report-Short Form; 95% CI = 95% Confidence Interval.

***Table S17:* Parameter estimates for change in posttraumatic stress symptoms over time relative to pre-deployment status with early sexual abuse score as covariate** (n=963).

|  | **Effect** | | **Interaction time x ETISR-SF sexual abuse** | |
| --- | --- | --- | --- | --- |
|  | Coefficient (95% CI) | P-value | Coefficient (95% CI) | P-value |
| Intercept (pre-deployment) | 26.58 (26.20 – 26.96) | <0.0001 |  |  |
| Δ 1 month^a^ | 0.96 (0.48 – 1.44) | <0.0001 | -0.45 (-1.39 – 0.48) | 0.343 |
| Δ 6 months^a^ | 1.10 (0.61 – 1.59) | <0.0001 | -0.92 (-1.81 – -0.03) | 0.042 |
| Δ 1 year^a^ | 0.39 (-0.15 – 0.93) | 0.153 | -0.71 (-1.61 – 0.19) | 0.122 |
| Δ 2 years^a^ | 0.16 (-0.39 – 0.72) | 0.566 | 0.14 (-0.96 – 1.23) | 0.808 |
| Δ 5 years^a^ | 1.64 (1.10 – 2.19) | <0.0001 | -0.41 (-1.38 – 0.56) | 0.411 |
| Δ 10 years^a^ | 1.02 (0.49 – 1.55) | <0.0001 | -1.25 (-2.21 – -0.29) | 0.011 |
| ETISR-SF sexual abuse score | 1.76 (1.01 – 2.51) | <0.0001 |  |  |

Note: ^a^ Δ indicates the difference relative to pre-deployment status; ETISR-SF = Early Trauma Inventory Self-Report-Short Form; 95% CI = 95% Confidence Interval.

**Latent trajectory growth mixture model analyses**

The latent trajectories were extracted from the data using a growth mixture model (GMM) in Mplus version 8.4. Missing data over time in the outcome variable was handled by full information maximum likelihood estimation (FIML). Time was modeled as the actual time-points occurred (i.e. -1; 1; 6; 12; 24; 60; 120), and was fixed between subjects. The model included a linear slope. We investigated one to six class solutions and used 5000 start values and 50000 iterations. First, a series of unconditional models were fitted (latent class growth analysis). Next, the models were re-fitted with a quadratic term for time to assess whether non-linear growth curves provided better fit to the data. Finally, the variances of the intercept were freed (growth mixture model). Due to non-convergence issues, the models did not allow a cubic term for time and free estimation of the time slope parameters. All models were compared on fit indices, entropy, class size, and interpretability (see Table S18 – 21).

***Table S18:* Fit indices for one to six solutions of the latent class growth analysis (LCGA).**

| **Fit indices** | **1 class** | **2 classes** | **3 classes** | **4 classes** | **5 classes** | **6 classes** |
| --- | --- | --- | --- | --- | --- | --- |
| AIC | 29133.52 | 27750.31 | 27377.08 | 27208.89 | 27054.77 | 26912.05 |
| BIC | 29177.35 | 27808.75 | 27450.13 | 27296.55 | 27157.04 | 27028.94 |
| Adj. BIC^a^ | 29148.77 | 27770.64 | 27402.49 | 27239.38 | 27090.34 | 26952.71 |
| Entropy | .. | 0.923 | 0.907 | 0.887 | 0.874 | 0.831 |
| Proportion of participants per class^b^ | .. | 0.141  0.859 | 0.796  0.022  0.182 | 0.195  0.054  0.748  0.003 | 0.003  0.211  0.027  0.712  0.048 | 0.060  0.003  0.026  0.073  0.188  0.649 |

*Note:* ^a^ BIC adjusted for sample size; ^b^ class proportions based on the estimated model; AIC = Akaike Information Criteria; BIC = Bayesian Information Criteria.

***Table S19:* Fit indices for one to six solutions of the latent class growth analysis (LCGA) including a quadratic term for time**.

| **Fit indices** | **1 class** | **2 classes** | **3 classes** | **4 classes** | **5 classes** | **6 classes** |
| --- | --- | --- | --- | --- | --- | --- |
| AIC | 29134.41 | 27735.04 | 27368.68 | 27171.03 | 27015.96 | 26899.04 |
| BIC | 29183.11 | 27803.22 | 27456.35 | 27278.17 | 27142.58 | 27045.14 |
| Adj. BIC^a^ | 29151.35 | 27758.76 | 27399.18 | 27208.30 | 27060.01 | 26949.86 |
| Entropy | .. | 0.919 | 0.882 | 0.888 | 0.849 | 0.832 |
| Proportion of participants per class^b^ | .. | 0.143  0.857 | 0.192  0.775  0.033 | 0.048  0.750  0.198  0.004 | 0.064  0.227  0.030  0.676  0.004 | 0.191  0.069  0.023  0.645  0.005  0.067 |

*Note:* ^a^ BIC adjusted for sample size; ^b^ class proportions based on the estimated model; AIC = Akaike Information Criteria; BIC = Bayesian Information Criteria.

***Table S20:* Fit indices for one to six solutions of growth mixture models (GMM).**

| **Fit indices** | **1 class** | **2 classes** | **3 classes** | **4 classes** | **5 classes** | **6 classes** |
| --- | --- | --- | --- | --- | --- | --- |
| AIC | 27633.53 | 27278.15 | 27102.49 | 26947.24 | 26816.43 | 26715.28 |
| BIC | 27682.23 | 27341.46 | 27180.41 | 27039.77 | 26923.57 | 26837.04 |
| Adj. BIC^a^ | 27650.47 | 27300.18 | 27129.60 | 26979.43 | 26853.67 | 26757.64 |
| Entropy | .. | 0.934 | 0.909 | 0.894 | 0.887 | 0.887 |
| Proportion of participants per class^b^ | .. | 0.054  0.946 | 0.013  0.920  0.068 | 0.062  0.067  0.011  0.860 | 0.096  0.020  0.814  0.058  0.011 | 0.020  0.100  0.009  0.792  0.075  0.004 |

*Note:* ^a^ BIC adjusted for sample size; ^b^ class proportions based on the estimated model; AIC = Akaike Information Criteria; BIC = Bayesian Information Criteria.

***Table S21:* Fit indices for one to six solutions of growth mixture models (GMM) including a quadratic term for time.**

| **Fit indices** | **1 class** | **2 classes** | **3 classes** | **4 classes** | **5 classes** | **6 classes** |
| --- | --- | --- | --- | --- | --- | --- |
| AIC | 27633.99 | 27280.36 | 27078.01 | 26920.19 | 26783.42 | 26674.48 |
| BIC | 27687.56 | 27353.41 | 27170.54 | 27032.20 | 26914.91 | 26825.45 |
| Adj. BIC^a^ | 27652.62 | 27305.77 | 27110.20 | 26969.15 | 26829.16 | 26726.99 |
| Entropy | .. | 0.932 | 0.900 | 0.890 | 0.882 | 0.860 |
| Proportion of participants per class^b^ | .. | 0.055  0.945 | 0.070  0.910  0.020 | 0.059  0.070  0.854  0.017 | 0.015  0.088  0.015  0.801  0.081 | 0.032  0.012  0.793  0.083  0.058  0.022 |

*Note:* ^a^ BIC adjusted for sample size; ^b^ class proportions based on the estimated model; AIC = Akaike Information Criteria; BIC = Bayesian Information Criteria.
